# Supplementary material for: The Potential Mechanisms behind Loperamide-Induced Cardiac Arrhythmias Associated with Human Abuse and Extreme Overdose
Source: Biomolecules. 2023 Sep 6;13(9):1355. doi: 10.3390/biom13091355 (PMC10527387; doi:10.3390/biom13091355)
Supplement: Supplementary file 1 [file biomolecules-13-01355-s001.zip › biomolecules-2579120-supplementary/Supplementary table S7.pdf]

Supplementary table 1. Electrophysiological effects of vehicle (n=6) and loperamide (n=6) on the Isolated Arterially Perfused Rabbit Left Ventricular Wedge.

	Vehicle					Loperamide				
Concentrations	**0.00%**	**0.00%**	**0.01%**	**0.03%**	**0.10%**	**0.1 µM**	**0.3 µM**	**1 µM**	**3 µM**	**10 µM**
**JT-interval**	1±1	2±2	2±2	3±3	3±3	3±2	6±4	3±5	-12±9*	-
**rTp-Te**	0±1	-1±2	-2±2	-3±4	-5±2	1±2	-6±4	-19±9*	-37±15*	-
**TdP Score**	0±0	0±0	0±0	0±0	-1±1	0±0	0±1	-1±0*	-1±0*	-1±0
**QRS rate dependency**	0±0	0±0	0±0	0±0	0±0	0±0	1±1	6±4*	30±23*	-
**JTp**	1±1	2±2	2±2	4±4	5±4	3±2	8±5*	8±6	-5±13	-

Data are expressed as % changes of the baseline value, and in MEAN±SD ; -: no measurable; * p<0.05
