# Supplementary material for: The Potential Mechanisms behind Loperamide-Induced Cardiac Arrhythmias Associated with Human Abuse and Extreme Overdose
Source: Biomolecules. 2023 Sep 6;13(9):1355. doi: 10.3390/biom13091355 (PMC10527387; doi:10.3390/biom13091355)
Supplement: Supplementary file 1 [file biomolecules-13-01355-s001.zip › biomolecules-2579120-supplementary/Supplementary table S8.pdf]

Supplementary table 2. Effects of vehicle (n=6) and loperamide (i.v. injection; n=6) in heart rate (HR), mean arterial blood pressure (MBP), and ECG parameters in Anesthetized Guinea-Pigs

	**Vehicle**						**Loperamide**					
**Doses**	0.5 ml/kg	0.5 ml/kg	0.5 ml/kg	0.5 ml/kg	0.5 ml/kg	0.5 ml/kg	0.16 mg/kg	0.32 mg/kg	0.63 mg/kg	1.25 mg/kg	2.5 mg/kg	5 mg/kg
**Time (min)**	0	15	30	45	60	75	0	15	30	45	60	75
**HR**	-3±5	-5±6	-9±4	-9±6	-10±6	-9±7	13±10*	12±11*	6±10*	-2±11	-16±13	-33±17*
**MBP**	-4±10	1±9	2±13	2±7	1±13	6±8	71±46*	53±30*	30±27	0±22	-38±19*	-55±22*
**QT-interval**	3±5	6±9	7±5	10±10	11±11	13±12	-8±8*	-7±9*	-2±9	6±11	27±17	58±26*
**Body Temperature**	-0.3±0.5	-1.1±0.7	-1.7±1	-2.7±1.4	-3±1.6	-3.4±1.8	-1±0*	-2±0*	-2.9±0.7	-3.4±0.5	-4±0.6	-4±0.8
**RR-interval**	2±7	5±7	8±5	9±8	10±9	10±9	-10±9*	-10±8*	-5±9*	4±13	24±21	57±40*

* p<0.05. HR: heart rate, MBP: mean arterial blood pressure.

Data are expressed as % changes of the baseline value and in Mean±SD.
