# Supplementary material for: The Potential Mechanisms behind Loperamide-Induced Cardiac Arrhythmias Associated with Human Abuse and Extreme Overdose
Source: Biomolecules. 2023 Sep 6;13(9):1355. doi: 10.3390/biom13091355 (PMC10527387; doi:10.3390/biom13091355)
Supplement: Supplementary file 1 [file biomolecules-13-01355-s001.zip › biomolecules-2579120-supplementary/Table 6 In-Silico Loperamide and hydroxazine.pdf]

Table S2. The cardiac electrophysiological effects of loperamide in-silico modeling.

Data are expressed as stimulated median values. FTPC: free therapeutic plasma concentration.

Parameters	Reference	Modeling concentration (fold: x over its FTPC of Loperamide)		
	0 µM (0x FTPC)	0.1 µM (400xFTPC)	0.25 µM (1000xFTPC)	0.35 µM (1400xFTPC)
Loperamide alone				
Peak Voltage. (mV)	43.2968	42.0121	41.55261	41.2189
RMP (mV)	-87.8071	-87.809	-87.8099	-87.8083
APD_{90} (ms)	357.97	396.05	462.58	503.545
APD_{50} (ms)	280.31	311	360	390.435
APD_{40} (ms)	253.68	279.18	318.37	341.34
$Triangulation_{90-40}$ (ms)	101.99	115.29	141.92	161.3
EAD (incidence: number of cells with EAD)	0	0	0	**1**
Loperamide and x1 FPTC of Hydroxyzine				
Peak Voltage. (mV)	43.2968	42.0196	41.5302	41.2652
RMP (mV)	-87.8073	-87.8084	-87.8085	-87.8086
APD_{90} (ms)	364.145	402.73	469.615	511.235
APD_{50} (ms)	285.47	315.16	364.71	395.875
APD_{40} (ms)	257.315	282.79	321.8	345.64
$Triangulation_{90-40}$ (ms)	104.75	118.42	145.555	165.695
EAD (incidence: number of cells with EAD)	0	0	0	**1**
Loperamide and x2 FPTC of Hydroxyzine				
Peak Voltage. (mV)	43.3123	42.0467	41.5725	41.3162
RMP (mV)	-87.8069	-87.8084	-87.807	-87.8089
APD_{90} (ms)	370.18	409.675	477.13	519.06
APD_{50} (ms)	289.58	319.695	369.095	400.98
APD_{40} (ms)	261.28	286.54	325.495	349.535
$Triangulation_{90-40}$ (ms)	107.47	121.68	148.84	169.815
EAD (incidence: number of cells with EAD)	0	0	**1**	**1**
Loperamide and x3 FPTC of Hydroxyzine				
Peak Voltage. (mV)	43.3237	42.09	41.6114	41.3459
RMP (mV)	-87.8063	-87.8081	-87.8069	-87.8111
APD_{90} (ms)	376.02	415.56	484.635	526.975
APD_{50} (ms)	293.58	323.955	373.94	405.345
APD_{40} (ms)	264.84	290.015	328.89	352.275
$Triangulation_{90-40}$ (ms)	110.06	124.59	152.54	174.365
EAD (incidence: number of cells with EAD)	0	0	**1**	**3**
